# Managerial Decision-Making of Nurses in Hospitals: creation and validation of a simulation scenario

**DOI:** 10.1590/1518-8345.6149.3768

**Published:** 2023-03-06

**Authors:** Nilva Maria Ribeiro, Laura Andrian Leal, Maria Verônica Ferrareze Ferreira, Lucieli Dias Pedreschi Chaves, Daniela Sarreta Ignácio, Silvia Helena Henriques

**Affiliations:** 1 Universidade de São Paulo, Escola de Enfermagem de Ribeirão Preto, PAHO/WHO Collaborating Centre for Nursing Research Development, Ribeirão Preto, SP, Brazil.

**Keywords:** Simulation, Nursing Education, Professional Skill, Decision-Making, Drug-Related Side Effects and Adverse Reactions, Patient Safety, Simulação, Educação em Enfermagem, Competência Profissional, Tomada de Decisões, Evento Adverso, Segurança do Paciente, Simulación, Educación en Enfermería, Competencia Profesional, Toma de Decisiones, Evento Adverso, Seguridad del Paciente

## Abstract

**Objective:**

to build and validate a clinical simulation scenario on hospital nurse managerial decision-making competence for undergraduate nursing students.

**Method:**

a descriptive and methodological study was carried out in a higher education institution, with the participation of 10 judges and five players. To do so, the conceptual simulation model proposed by Jeffries and standards of the International Nursing Association for Clinical Simulation and Learning were used to prepare the scenario and the checklist.

**Results:**

the scenario was called “Managerial decision-making of nurses in the face of adverse events in a hospital”. The scenario script and checklist were built for validation. The checklist was face- and content-validated. Afterward, judges used the checklist to validate the scenario, which, in its final version, was composed of Prebriefing (seven items), Scenario in Action (18 items) and Debriefing (seven items).

**Conclusion:**

the scenario proved to be a teaching strategy that anticipates the reality of future nurses, bringing them the self-confidence to perform their activities and helping them to act critically and reflectively during decision-making processes.

## Resumo

**Objetivo:**

construir e validar um cenário de simulação clínica sobre a competência tomada de decisão gerencial do enfermeiro hospitalar para estudantes de graduação em enfermagem.

**Método:**

estudo descritivo e metodológico realizado em uma instituição de ensino superior, com a participação de 10 juízes e cinco atores. Utilizou-se o modelo conceitual de simulação proposto por Jeffries e guias padronizadas da *International Nursing Association for Clinical Simulation in Learning* para a elaboração do cenário e do *checklist*.

**Resultados:**

o cenário ficou denominado como “Tomada de decisão gerencial do enfermeiro diante de eventos adversos no contexto hospitalar”. Construiu-se o *script* do cenário e o *checklist* para a sua validação. Realizou-se validação de face e conteúdo do *checklist*. Posteriormente, juízes de posse do *checklist*, validaram o cenário que, em sua versão final, ficou composto por *Prebriefing* (sete itens), Cenário em Ação (18 itens) e *Debriefing* (sete itens).

**Conclusão:**

o cenário mostrou-se uma estratégia de ensino capaz de antecipar a realidade do futuro enfermeiro, trazendo autoconfiança na execução de suas atividades, contribuindo para agir de forma crítica e reflexiva durante o processo de tomada de decisão.

## Resumen

**Objetivo:**

construir y validar un escenario de simulación clínica sobre la competencia en la toma de decisiones gerenciales del enfermero hospitalario para estudiantes del grado en enfermería.

**Método:**

estudio descriptivo y metodológico realizado en una institución de educación superior, con la participación de 10 jueces y cinco actores. Se utilizó el modelo conceptual de simulación propuesto por Jeffries y guías estandarizadas de la *International Nursing Association for Clinical Simulation in Learning* para la elaboración del escenario y del *checklist.*

**Resultados:**

el escenario se denominó “Toma de decisiones gerenciales del enfermero ante eventos adversos en el contexto hospitalario”. Se construyó el *script* del escenario y el *checklist* para su validación*.* Se realizó la validez aparente y el contenido del *checklist*. Posteriormente, los jueces en posesión del *checklist*, validaron el escenario que, en su versión final, quedó compuesto por *Prebriefing* (siete ítems), Escenario en Acción (18 ítems) y *Debriefing* (siete ítems).

**Conclusión:**

el escenario demostró ser una estrategia de enseñanza capaz de anticipar la realidad del futuro enfermero, trayendo autoconfianza en la ejecución de sus actividades, contribuyendo para actuar de forma crítica y reflexiva durante el proceso de toma de decisiones.

**Highlights**

(1) Innovative study in the teaching-learning process of nursing management.

(2) A validated clinical scenario on the managerial decision-making of nurses was applied.

(3) Expanded view of nursing work process through the use of simulation.

(4) Professional skill development and learning deficit reduction.

(5) An opportunity to recognize adverse events in hospitals was presented.

## Introduction

Scientific evidence has shown that a hospital is a place of health care that requires trained nurses to deal with different situations. In hospitals, the deficit of professional managerial skills is associated with factors that negatively affect the outcomes of users^1^. In this scenario, nurses have played the role of manager of their team, thus requiring different knowledge to meet the demands of the institution, thus promoting excellence in care.

Following this line of thought, the development of specific professional skills in nursing management should be considered for the daily professional practice of nurses in a hospital unit^2^. Given this, the training of these professionals should include discussions also focused on management issues. The training focused on leadership, management, communication, decision-making and ongoing education is known to help professionals achieve knowledge, skills and attitudes, that is, managerial skills for working in the health sector^3^.

In this study, decision-making is highlighted as a managerial competence of nurses, which can be learned during academic training, in addition to the theoretical field, carrying out a practical approach through realistic simulation, which is considered a methodological strategy that benefits students during undergraduate studies^4^. Stimulating clinical reasoning and decision-making to perform assertive procedures through simulations improves adverse event prevention, ensuring better nursing care^5^. Certain actions or resolutions, to make a decision, must be chosen based on prior knowledge, since the hospital scenario requires initiatives based on the level of complexity of user care, especially in a short time for assertive choices and deliberations.

Clinical simulation applied to nursing students should favor the development of skills for managerial decision-making, making them more active in the face of existing needs during nursing care, as observed in a study with 233 nursing students in central Portugal^6^. Through curricular stimuli required by ministerial decrees, training centers have adopted innovative pedagogical tools, such as realistic simulations for more efficient learning^7^ ^-^ ^9^.

Given the above, this study has the following guiding questions: what are the stages of construction and validation of a clinical simulation scenario on managerial decision-making in hospital nurses? and how is the final version of such a scenario to be applied to undergraduate nursing students?

Valid and reliable simulated clinical scenarios should be applied to students through the integration of systematic approaches based on evidence that the participants are aware of^10^, considering the opinion of judges/experts to ensure alignment with existing good practices. This is because previous systematic reviews have shown the effectiveness of simulation as a teaching and learning strategy, important for the development of clinical competence and academic performance^11^ ^-^ ^12^.

This study aimed to build and validate a clinical simulation scenario on hospital nurse managerial decision-making competence for undergraduate nursing students. 

## Method

### Study type

This study is defined as descriptive and methodological and made use of the conceptual model of simulation proposed by Jeffries as a methodological framework. In this model, different elements make up a simulation, such as a theme identification, simulation goals, participants, simulation scenarios and debriefing^13^. Furthermore, this study followed the best-practice standards for simulations published by the International Nursing Association for Clinical Simulation and Learning, with the prebriefing, scenario, and debriefing phases for scenario development in addition to the checklist^14^.

Data structuring followed recommendations in the revised Standards for Quality Improvement Reporting Excellence (SQUIRE 2) published in the Equator Network Library^15^.

### Data collection location 

Data were collected at the Nursing Practice Simulation Center of a Higher Education Institution (HEI) located in the city of Ribeirão Preto - São Paulo State, Brazil. The institution offers two undergraduate nursing courses, one for a Bachelor’s degree and another for a Bachelor’s and a Teaching degree.

### Period

Data collection took place between May 2019 and February 2020.

### Population

Judges participated in the elaboration and validation of the scenario. Moreover, nurses and graduate nursing students participated as players to represent the respective scenario.

### Selection criteria

The judges were selected by consulting the *Curriculum Vitae* of researchers, considering academic degrees, years of experience in clinical practice, research carried out in the area of interest of the study, published articles on the subject and participation in events in the field. 

### Participants

Ten judges took part in the study. Five participated in the online validation of the checklist and the other five participated in the face-to-face validation of the scenario. These participants comprised professional nurses, teachers and experts in the field of clinical simulation. An odd number of experts, as well as a minimum of three judges, is recommended to assess items related to equivalence and agreement of responses. The experts must have experience and technical/scientific knowledge, besides being able to analyze and judge items related to the scenario, which were selected by convenience sampling^16^. 

Two graduate students, a nurse from the Simulation Center of the selected institution, and two researchers/authors of this research participated in the face-to-face validation of the scenario. These players were chosen based on their previous experience in other simulation activities.

### Study variables 

There is none.

### Instruments used in information collection

Initially, a script of the scenario was prepared and contained information about it. Then, the authors built an instrument to be used by judges, called a checklist. This tool had four domains: prebriefing, scenario in action, debriefing and general assessment for later calculation of the Content Validity Index (CVI). 

In a second moment, the face-to-face validation of the scenario was performed during a simulation where the judges used a validated checklist to signal whether actions were taken or not, as they happened, in addition to making suggestions.

### Data collection 

In the first stage, the checklist used was sent by e-mail to five judges, along with the scenario script, requesting an evaluation and return within 15 days. With this, the researchers could calculate the CVI, using a cutoff point of 0.80 as a minimum to characterize an item as valid. Three aspects were considered for the face-and-content validity of the checklist, namely: clarity, relevance and appearance. A five-point ordinal Likert scale was used, assigning values from one to five for each item, in which: (5) I totally agree, (4) I agree, (3) neither agree nor disagree, (2) disagree, (1) strongly disagree. 

In the second stage, the scenario was validated face-to-face by five other expert judges on the scheduled day and time at the Nursing Practice Simulation Center of the public HEI selected. The checklist was made available to the judges, containing prebriefing, scenario in action and debriefing for scenario validation. Five players participated in the representation of the scenario, with the nurse from the HEI Simulation Center playing the role of doctor and nurse, two graduate nursing students, one as a patient/client and the other as a nurse, while the scenario was conducted by two researchers/research authors. The players were explained the scenario stages and, before their performance, they were given the checklist so that they could become familiar with each simulation stage. Guidelines were also provided on the clinical case, research goals, materials and equipment available and presentation of the environment.

### Data processing and analysis

Data related to the face and content validation of the instrument was analyzed by calculating the Content Validity Index (CVI)^17^, which shows the congruence of the expert judges’ opinions through the proportion of agreement on the scenario simulation questions that had been validated.

The CVI was calculated considering the acceptable agreement rate among the members of the expert committee, which must be at least 80% and, preferably, above 90%^18^.

AC1 statistic was used to assess the agreement between the judges for each item describing the scenario in the checklist, regarding the criteria of clarity, appearance and relevance^19^.

The agreement analysis was performed using the R Core Team software^20^ version 3.5.3, which can be downloaded free of charge from the website: www.r-project.org. All analyses were carried out adopting a significance level of 5% (alpha = 0.05).

### Ethical aspects

In compliance with the requirements of Resolution 466/2012 of the National Health Council (CNS, in Portuguese), which regulates the rules for conducting research involving human beings, all participants in this study signed a Free and Informed Consent term (FIC). The study was approved by the Research Ethics Committee of the proposing institution under protocol CAAE n° 01435418.1.0000.5393. 

## Results

A scenario script was built according to the methodological framework to meet the objective of the study. The script was composed of the following topics: general and specific goals, estimated time, prebriefing, scenario development, evaluation, expected actions, evolution, debriefing, environment, participants, materials and medicines.

To validate the scenario, a checklist was constructed and validated (an instrument that was used by the judges during the scenario validation). 

At first, for validation, the checklist had four domains, namely: domain 1 - Prebriefing (10 items), domain 2 - Scenario in Action (18 items), domain 3 - Debriefing (7 items), and domain 4 - General Assessment (4 items), totaling 39 items. Thus, in domains 1, 2 and 3, the judges had to evaluate each item considering clarity, relevance and appearance. 

The prebriefing items comprised preparatory instructions for scenario development, for example, selection of participants, presentation of the environment where the scene would take place, in addition to presentation of the scenario objectives and clinical case. The scenario in action was the actual time of simulation, in which a situation is created and replicated to be developed by the student, as close to reality as possible. The debriefing domain, on the other hand, takes place after the simulation, with the facilitator encouraging participants to reflect on the execution of the scenario, seeking to improve or confirm the practice performed, stimulating communication, trust, and confidentiality among those involved, who can give their opinion on issues involving the simulation experienced. Finally, in the general assessment domain, a space was made available for the judges’ suggestions.

To present the final version of the checklist and CVI calculation, improving understanding, we opted to divide it according to Figures 1, 2 and 3. 

As shown in Figure 1, considering the 10 items initially evaluated for clarity, relevance, and appearance in the prebriefing, 73.33% of them had a CVI above 80%, ranging from 60 to 100%. CVIs were below 80% on two items for appearance, three for agreement, and two for relevance. Still, according to the average CVI (a validated index of agreement between the judges for the total content of each question), four items had an average CVI of less than 80%, ranging from 60 to 73.3%. Therefore, to adapt according to the judges’ criteria, we decided to restructure the instrument, whose prebriefing domain was reduced from 10 to seven items.

Next, Figure 1 shows the prebriefing domain of the final version of the instrument, which was used by the judges during scenario validation.

**Figure 1:** Final version of the prebriefing domain of the simulated scenario checklist. Ribeirão Preto, SP, Brazil, 2019-2020

Domain description	Item	Specification
Prebriefing	1	Facilitator introduces himself to students
	2	Facilitator offers content about managerial decision-making in nursing
	3	Facilitator establishes a confidentiality agreement with the entire group
	4	Facilitator invites two students to participate in the scenario
	5	Facilitator presents the scenario’s general objectives for all players and students
	6	Facilitator presents maximum scenario time to participating players and students
	7	The scenario is presented to participating students, providing time for familiarization with the environment and material resources available to players/students

Regarding the Scenario in Action domain, shown in Figure 2, in the appearance criterion, among the 18 items evaluated, only one item had a CVI of 60% and the others had a CVI above 80%. The clarity criterion assessment had a much higher proportion of items with results below 80%, totaling seven ranging from 40 to 60%. Finally, two items received a CVI of 60% in the relevant domain, with the others having a CVI above 80%.

Therefore, when analyzing the average CVI of the judges, all items of the appearance criterion obtained values between 4.2 and 5, indicating a very favorable appearance. In the agreement criterion, only two items obtained values below 4 (i.e., 3.4 and 3.8), which led to consider the need to be reformulated, while the others showed good agreement. And for the relevance criterion, one of the items obtained a value of 3.8 and also underwent reformulation, whereas the other 17 items showed good relevance. 

To improve the scenario, the guidelines issued by the judges were considered, making it clearer and easier to understand and facilitating its replication for students. Thus, some items were reformulated and, in the end, for the Scenario in Action domain, 18 items remained, as shown in Figure 2.

**Figure 2:** Final version of the “Scenario in Action” domain of the simulated scenario checklist. Ribeirão Preto, SP, Brazil, 2019-2020

Domain description	Item	Specification
Scenario in action	1	Facilitator presents the clinical case to all students and players, explaining the activity to be carried out and detailing the situation
	2	Participating students, in the role of nurse and nursing technician on the afternoon shift, receive the handover from the morning shift nurse. Afterward, this one leaves the scene
	3	Nurse and nursing technician (students) who were on duty wash their hands
	4	Nurse and nursing technician (students) take the patient’s chart and go to the patient’s room
	5	Nurse and nursing technician (students) introduce themselves to the patient
	6	Nurse and nursing technician (students) find an antihypertensive drug on the bedside table
	7	Nurse (student) evaluates the general conditions of the patient (interview and physical examination)
	8	Nurse (student) checks the patient’s vital signs or asks the nursing technician (student) to perform this activity, noting changes in blood pressure
	9	Nurse (student) finds it necessary to contact the nurse on the previous shift and the physician on duty about the intercurrence
	10	Nurse (student) calls the nurse in charge of the morning shift (the one who handed the duty over) and seeks to know more information about the morning shift
	11	Nurse (student) calls the physician on duty and tells them that the antihypertensive medication was not administered at the prescribed time and reports the patient’s vital signs
	12	Physician on duty requests to change the antihypertensive administration schedule
	13	Nurse (student) asks the nursing technician (student) to administer medications according to the medical prescription
	14	Nurse (student) asks the nursing technician (student) to strictly control the patient’s blood pressure
	15	Nurses (student) and nursing technician (student) double-check the antihypertensive medication administered at the time of the medical prescription
	16	Nurse (student) performs nursing evolution reporting the intercurrence
	17	Nurse (student) tells the patient about the occurrence and raises awareness about the importance of medication at the right time
	18	Nurse (student) guides nursing technician (student) on safe practices in the medication administration process

When analyzing the last domain, the debriefing, which is composed of seven items, it was observed that the criteria of appearance and relevance had CVI above 80% for all items. For the agreement criterion, only one item had a CVI of 60%, while the others had values above 80%. Overall, CVI remained between four and five, indicating favorable appearance, agreement and relevance. Based on these results, the debriefing domain structure had no changes, remaining as in the first version of the instrument (Figure 3).

**Figure 3:** Final version of the simulated scenario checklist debriefing domain. Ribeirão Preto, SP, Brazil, 2019-2020

Domain description	Item	Specification
Debriefing	1	Facilitator reiterates the simulation purpose to the group
	2	Facilitator invites players to reflect on how they felt acting in the scenario and identify the type of scenario proposed according to the theme
	3	Facilitator encourages viewers to raise positive aspects of student’s performances in the scenario
	4	Facilitator encourages participants to reflect on potential practices different from those performed (starting with those who participated in the scenario)
	5	Facilitator discourages destructive criticism if any
	6	Facilitator stimulates students on important aspects of decision-making competence, articulating theory and practice
	7	Facilitator encourages students to suggest other decision-making possibilities that were not presented in the scenario performed

The fourth domain, present in the initial version and referring to general assessment (4 items), was removed from the final version, thus, the scenario checklist had 32 items.

Checklist face and content were validated after all adjustments and returned to judges with positive final positioning, followed by face-to-face validation of the simulated scenario. In this validation, another five decision-making expert judges acted as players. Thus, they validated the simulation scenario by indicating in the checklist whether items occurred in each stage, writing “positive, negative, or partly”. Still, there was room for suggestions, if needed. 

In the face-to-face scenario validation, in the prebriefing domain, judges considered that actions during the scenario development corresponded to the checklist, noting them as performed. One of the judges suggested that the facilitator would guide the students on how much time they would have to familiarize themselves with the environment and material resources available in the laboratory for players/students. Greater emphasis on scenario presentation by the tutor to the scenario players and listeners was also recommended. Furthermore, the importance of including length of stay in the presentation of a clinical case was highlighted. 

In the Scenario in Action domain, judges emphasized that students must know the simulated environment in detail so as not to get lost when performing tasks. For instance, starting the scenario with hand washing and measuring vital signs, especially blood pressure, to detect changes and initiate the decision-making process. One of the judges suggested that when the student (nurse) was calling the doctor (actor), instructions received should be directed to the second student (nursing technician) aloud, while still on the phone with the doctor. A printed document was also suggested to be made available to record scenario actions in the patient’s chart, as well as use a pain scale while assessing the patient’s general conditions.

In the debriefing domain, judges emphasized the need to ask participants about potential practices other than those performed in the scenario. Judges also suggested considering the patient’s emotional aspects during discussions.

Thus, the final version of the simulation scenario to be applied to nursing students includes the three domains: prebriefing, scenario in action and debriefing, as well as their respective elements (Figure 4 and Figure 5).

**Figure 4:** Simulated scenario script: “Managerial decision-making by nurses in the face of adverse events in a hospital” - Part 1. Ribeirão Preto, SP, 2019-2020

Scenario: Managerial decision-making by nurses in the face of adverse events in a hospital
Persons in charge: The authors
Target audience: Students enrolled in the fourth and fifth years of undergraduate nursing
General goals: Provide nursing care to a hypertensive patient diagnosed with community pneumonia and hospitalized in a Medical Clinic unit, focusing on managerial decision-making in the face of an adverse event.
Specific goals: Evaluate the patient’s general conditions (interview and physical examination). Implement safe medication administration practices and verify the nine rights of medication administration#. Use managerial decision-making in the face of the absence of antihypertensive administration at the prescribed time. Communicate with the nurse on duty and the responsible for the patient to understand what happened. Inform the doctor about the fact and check for potential antihypertensive medication schedule adjustments. Carry out nursing records in the patient’s medical record.
Estimated time: Prebriefing: five minutes; Scenario: 10 minutes; Debriefing: 20 minutes
Prebriefing: Facilitator introduces himself to students, establishes a confidentiality agreement and invites two students to participate in the scenario, presents general goals of the scenario to all participants, presents maximum time for the scenario execution, making time available for participants to recognize the environment and material resources.
Development of the scenario in action:
Facilitator presents the clinical case and details the situation to everyone. Patient J.C.M, 65 years old, on the third day of admission to the medical clinic with a diagnosis of community-acquired pneumonia and systemic arterial hypertension (SAH). The lifestyle factors smoking and sedentarism were referred to. She is conscious, and oriented and does not require help to walk. There is a medical prescription and nursing evolution in the patient’s chart.
The unit’s daily staff includes one nursing technician (nursing student) responsible for the direct care of the patient whose initials are J.C.M., it also includes one nurse (student) responsible for the hospital unit, in addition to a physician on duty who can be reached by phone.
At 1:30 pm, the afternoon shift nurse (represented by the nursing student) receives the shift from the morning shift nurse.
Shift Handover: Good afternoon! Patient Juliana Correia Martins is in room 102, bed 1. She is on her third day of hospitalization and diagnosed with community pneumonia. She is conscious, oriented, communicative, and can walk by herself. She is on medication prescribed by the doctor. A little while ago, the patient complained of a headache, but there was an intercurrence during the shift and I could not evaluate her, so I suggest you start visiting her. Have a good shift!
Evaluation: A - Pervious airway; B - Breathing ambient air; PO_2_S^*^: 95%; RR^†^: 24 RPM^‡^; C - BP^§^: 70/100 mmHg^ǁ^ and HR^¶^: 95 BPM^**^;
D - Communicative, oriented and anxious; E - Peripheral venous access on the back of right hand salinized. Normothermia (Tº^††^: 36.5ºC).

*PO_2_S = Peripheral oxygen saturation; ^†^RR = Respiration rate; ^‡^RPM = Respirations *per* minute; ^§^BP = Blood pressure; ^||^mmHg = Millimeters of mercury; ^¶^HR = Heart rate; **BPM = Beats *per* minute; ^††^T = Temperature; ^#^Check the nine rights of medication administration: right patient, right drug, drug compatibility, patient guidance, right to refuse drug, correct note, right dose, right route and the right time^21^

**Figure 5:** Simulated scenario script: “Managerial decision-making by nurses in the face of adverse events in a hospital” - Part 2. Ribeirão Preto, SP, 2019-2020

*Expected actions:*
A: Assess airways; B: Check RR^*^, elevate the head of the bed; C: Perform non-invasive blood pressure measurement and HR check^†^; D: Assess the level of consciousness; E: Check peripheral venous access and measure body temperature. Others: Assess the patient’s general conditions (interview and physical examination); Implement safe medication administration practices and verify the nine rights of medication administration#; Contact the previous shift nurse responsible for the patient for information on the adverse even through a telephone call; Communicate the fact to the doctor (who is reached by phone), check for potential adjustment of antihypertensive medication schedule and administer the medication according to medical advice, double-checking the medical record; Advise the patient about the complication and which course of action will be taken; Record the activities performed in the medical record.
Evolution: Patient presents normalization of vital signs and no pain complaints.
*Debriefing:*
*1. Questions to Players:*
How did you feel (feeling/emotion) acting in the scenario? Could you describe the scenario experienced? What were the positive points? What would you do differently? What points could be improved? What will you take with you in your life? What learning did you get? What is the professional competence developed in this scenario? After participating in this scenario, what do you think about the exercise of decision-making competence?
*2. Questions to Observers:*
What are the positive points observed in this service? What learning did you get from it?
Summary: The discussion should permeate the nurse’s decision-making steps: assessment of patient’s general condition and priority care to its immediate needs, identification of team members responsible for patient care in both shifts, communication with physician on duty, medication administration, checking prescription and adjusting medication time and guidance of other team members. For a professional practice framework, the role of a nurse’s managerial decision-making competence should be highlighted, as well as the need to associate theory and practice; in addition, pathological processes and their symptoms should be well known to efficiently identify which decision-making to adopt. Carry out permanent education actions to the team on safe medication administration practices.
Environment: Medical clinic sector.
*Participants:*
1 student in the role of the nurse;
1 player in the role of the patient;
1 student in the role of the nursing technician;
1 player in the role of the nurse and doctor;
1 player in the role of the nurse.
Materials and equipment: patient’s medication tray (antihypertensive); hospital bed with ward sheets; cup and jar containing water; sphygmomanometer; stethoscope; bed identification card; trash can; peripheral venous access equipment; bedside table; portable oximeter; wig; patient identification bracelet; clock; telephone, clinical thermometer and medical records (history, medical diagnosis, prescription, nursing evolution, and vital signs).	

*RR = Respiratory rate; ^†^HR = Heart rate; ^#^Check the nine rights of medication administration: right patient, right drug, drug compatibility, patient guidance, right to refuse drug, correct note, right dose, right route and the right time^21^

## Discussion

To build scenarios, systematization and careful planning should be considered, in addition to the use of tools to instrumentalize professor/facilitator activity^7^ ^-^ ^10^.

The proposed scenario brought a real clinical situation, seeking to emphasize the importance of managerial decision-making and its practice by undergraduate nursing students. This way, as future professionals, they will be able to act with critical and reflective thinking about the best practice to be established in the work environment. Therefore, the importance of a previous experience through validated clinical scenarios is here emphasized.

Results highlighted the steps required to build a clinical simulation scenario. For greater reliability of the scenario validity, the checklist, an instrument used to validate the scenario, underwent face and content validation. The results obtained with the validation were positive and suggestions from judges brought greater quality to the scenario, making it closer to reality.

In the face validation, the understanding and acceptance of the instrument by the judges were verified, seeking to ensure the understanding by the participants and clear doubts that could hinder the achievement of goals. Face validation involves understanding and accepting the instrument by subjects^22^. In turn, the content validity verifies whether all domain questions are representative of the universe of all questions to be asked about the theme, allowing for verification of whether the instrument contains all relevant components and domains related to the phenomenon^23^. 

The first built scenario domain (prebriefing) brought simulated environment guidelines, available resources, and simulation strategy. This must be presented clearly and objectively to favor communication and a relationship of trust among the participants. At this time, the participants were also oriented, before starting the simulation itself. This corroborates the findings in the literature, in which during prebriefing, all orientation and preparation of participants must be carried out to assimilate the simulation proposal. Some items are suggested at this stage, such as guidance on simulation goals, equipment, the environment, mannequins if used, duration time and scenario, among others^13^.

For that purpose, a favorable environment must be created, wherein errors are considered opportunities to improve, exercising the simulated practice; therefore, at this stage, strategies to develop greater interaction between participants should be sought, increasing their participation^24^.

During the scenario development stage, participants had to be informed about the progress of the clinical case, as well as the user’s conditions and previous history so that they could understand the care priorities. Thus, participants in clinical simulations must be guided in a planned way^25^. 

The last stage of scenario development, the debriefing, was the domain that received the best evaluation from judges. This step is essential for students to apply theoretical learning in practice, enabling them to solve problems and make the most appropriate clinical decisions^26^. Thus, learning decision-making competence is related to acting and leads nursing students to acquire knowledge and develop other global competencies, leading to a practice of excellence^27^. 

During the debriefing, the discussion addressed the decision-making stages of nurses, as follows: assessment of the patient’s general conditions and priority care for her immediate needs, identification of team members responsible for patient care in the two shifts, whether there was communication with the physician on duty, prescription checking and medication schedule adjustment, medication administration and guidance of the other team members. Moreover, the importance of decision-making competence of nurses for professional practice and the need to associate theory and practice should be highlighted, as well as their knowledge of pathological processes and respective symptoms, so that these professionals could be efficient in problem-solving.

 Finally, at this stage, the nursing team should be permanently educated on safe medication administration practices since they are known to be a concern among the care activities, with vulnerabilities being identified in the process, making it inappropriate and hence undesirable^28^. 

Realistic simulations must go beyond technical skills but include non-technical skills^29^, which include managerial skills such as decision-making by nurses, the subject of this study. Although managerial skills are recognized as indispensable for nurses, they are not effectively developed at work. In this way, institutional education strategies and improvements in academic training should be a priority and can be developed by changes in the undergraduate curricula, including alternative and innovative teaching methods, such as simulations, to make students critical in terms of managerial decision-making^1^.

In this regard, scenarios must be well developed for a successful application, promoting the development of competencies, positive experiences with students, stimulating decision-making, and effective problem-solving^10^. Still, the scenario developed in this study can make nursing undergraduate students able to develop permanent education actions during immersion in practice.

Clinical simulation brings advantages to undergraduate students. Unlike traditional education, the simulations allow bringing theory and practice together, developing psychomotor skills and encouraging communication among participants. Knowledge acquired in simulations is believed to be more difficult to forget, thus improving students’ professional performance. Simulations also increase student confidence and satisfaction by being conducted in a controlled and protected environment. This environment seeks to approach scenarios closer to reality, allowing error correction. Therefore, simulation is an important teaching method in undergraduate healthcare programs, especially in nursing^30^.

Because of the above, it is emphasized that instruments and clinical scenarios must be validated with experts on the subject, that is, specialists must present agreement or disagreement for each stage of the scenario before its use.

The approach to undergraduate teaching methods must be constant and standardized, and simulations must always seek new knowledge. The use of simulation in nursing teaching-learning contributes to the development of cognitive and technical skills capable of transforming the teaching process and, consequently, the professional training of nurses^31^.

The results of education through simulation are positive, but how simulations are structured and conducted is variable, showing that validated instruments are little used^13^. Developed scenarios should be validated because, if they are little clear or reliable, their replication by other educators and researchers is hindered^32^. There are countless benefits of implementing teaching strategies by active methods, in which high technology is not always necessary to ensure learning success. This is because the financial difficulties of educational institutions must be overcome so that simulations could achieve good results^33^.

As it was developed and validated in a specific hospital area, the scenario in this study was identified as limited, restricting its use in other health care sectors although nurse managerial decision-making is one of their essential skills in the work process.

Concerning nursing, this study brings advances to scientific knowledge since it is innovative. Here, undergraduate nursing courses have the opportunity to use with students a validated scenario focused on managerial decision-making and thus optimize their knowledge based on a realistic experience. Furthermore, this scenario can contribute to an expanded view of the nursing work process, which goes beyond assistance, bringing decision-making as a process that requires extensive knowledge to solve it.

## Conclusion

Managerial decision-making is a competence of hospital nurses in their daily work, which makes them professionals in great demand to solve problems in the unit and care provided to users. In this context, the use of clinical simulation as a teaching and learning strategy for nursing students, anticipating probable real situations that involve decision-making, provides relevant skills for their professional practice as future nurses. 

In this direction, the clinical simulation scenario proposed in this study included the steps required for its construction and validation. It, thus, has become viable and can be used by higher education institutions, to develop managerial decision-making competence for nursing students. 
